# Long-term outcome of lamivudine/dolutegravir dual therapy in HIV-infected, virologically suppressed patients

**DOI:** 10.1186/s12879-022-07769-6

**Published:** 2022-10-12

**Authors:** Franco Maggiolo, Roberto Gulminetti, Layla Pagnucco, Margherita Digaetano, Adriana Cervo, Daniela Valenti, Annapaola Callegaro, Cristina Mussini

**Affiliations:** 1 Division of Infectious Diseases, ASST Papa Giovanni XXIII, Bergamo, Italy; 2 Division of Infectious Diseases, Fondazione IRCCS Policlinico San Matteo, Pavia, Italy; 3 Division of Infectious Diseases, University of Modena, Modena, Italy; 4 Microbiology and Virology Laboratory, ASST Papa Giovanni XXIII, Bergamo, Italy; 5 Fondazione FROM, Bergamo, Italy

**Keywords:** Dual ART, Dolutegravir, Lamivudine, Switch, Simplification, Long-term follow-up, Cohort

## Abstract

**Background:**

The use of DTG-containing two-drug regimens is one of the most promising solutions to the need to ease the management of HIV treatment without harming its efficacy and safety. We report long- term results in patients switched, while virologically suppressed, to the combination of dolutegravir (DTG) plus lamivudine (3TC).

**Methods:**

This is a prospective, clinical, uncontrolled cohort enrolling ART-experienced people living with HIV (PLWH) with HIV-RNA < 50 copies/ml for 6 months or longer, negative hepatitis B virus surface antigen, and without known M184V/I mutations. Kaplan-Meiers curves are used to describe persistency of virological suppression on therapy and a Cox regression model to evaluate baseline characteristics and the risk of stopping therapy.

**Results:**

218 individuals switched their regimen since 2015. The mean estimated follow-up was of 64.3 months (95% CI 61.3–67.3) for approximately 1000 patient/years. After 5 years of follow-up, 77.1% were still on the DTG-3TC combination. No virologic failure was detected throughout the whole study period, and only 15 subjects presented single isolated viral blips above 50 copies/ml. Most patients stopped therapy because of reasons unrelated to study drugs (lost to follow-up; patients’ decision; moved to other Centers), but due to the unselected nature of the casuistry; 11 subjects died in the 5 years of follow-up mostly because of pre-existing co-morbidities (6 neoplastic diseases and 2 end-stage liver disease). The median baseline CD4 count was 669 cells/mcl (IQR 483–927). After 5 years it raised to 899 cells/mcl (IQR 646–1160) (P < 0.001) without a significant change of CD8 counts that lowered from 767 cells/mcl (IQR 532–1034) to 683 cells/mcl (IQR 538–988). Consequently, the CD4/CD8 ratio varied from 0.93 (IQR 0.60–1.30) to 1.15 (IQR 0.77–1.45) (P < 0.0001). A non-significant (P = 0.320) increment of mean creatinine, 0.06 mg/dl in magnitude, was observed over the whole follow-up.

**Conclusion:**

These long-term results over 5 years reinforce the durability and good tolerability of DTG-3TC. Our results continue to support the recommended switch use of this 2DR as a well-accepted treatment option for ART-experienced PLWH.

## Introduction

Modern antiretroviral therapy (ART) allows for several treatment options with high efficacy and tolerability, available for both ART-naïve and -experienced people living with HIV (PLWH) [1, 2]. The advent of an integrase inhibitor (INSTI) as dolutegravir (DTG), with high potency and high genetic barrier has allowed to expand strategies for initiating and simplifying ART [3–5]. Moreover, new ART strategies with fewer drugs have been recently introduced in clinical practice and are becoming widely used worldwide [3–8], leading to a scenario where therapies can be increasingly tailored to combine efficacy and tolerability with better ease of intake for PLWH, who consequently may experience an additional benefit in terms of quality of life. Furthermore, the opportunity for single tablet regimens seems to satisfy patient preferences and improve adherence [9–13]. The use of DTG-containing two-drug regimens (2DR), and, in particular, of DTG plus lamivudine (3TC), is one of the most promising solutions to the need to ease the management of HIV treatment without harming its efficacy and safety.

As most of these strategies were introduced in recent years, long-term durability studies are needed to definitively establish the future role of less-drug regimens. We report a prospective, clinical, uncontrolled experience on patients switched, while virologically suppressed, to the combination of dolutegravir plus lamivudine.

## Materials and methods

The cohort started in 2015. In 2017, our study group published the short-term results of the cohort 24 weeks after the switch to DTG/3TC combination. The inclusion criteria to build the cohort and the methods applied were described in detail in the previous study [14]. Briefly, we considered for inclusion in this cohort only patients that at the moment of therapeutic switch had a HIV-RNA < 50 copies/ml for 6 months or longer, were negative for hepatitis B virus surface antigen, and were on a stable ART (for > 6 months). Generally, ART was based on a nucleoside backbone plus a third anchor agent, or, in a few cases on other complex regimens. Further, only patients with no previous resistance mutations to either integrase inhibitors or lamivudine were included.

Resistance had to be determined by genotypic analysis before the start of ART or afterward in the occasion of viral blips before the current regimen was started. Patients were not included if they had a virological failure following their last genotypic test. Subjects were switched to a dual combination of dolutegravir (50 mg once daily) plus lamivudine (300 mg once daily).

The possibility of the switch to DTG and 3TC was previously discussed individually with each patient, among other available drug regimens and according to available data [2, 15, 16]; in all patients, the decision to switch therapy was taken on clinical grounds as they presented a clinically relevant reason, either because of concomitant diseases, altered laboratory tests, drug adverse events or risk of drug-to-drug interactions.

Since Italian guidelines considered DTG/3TC combination a well-established alternative option in ART management [17], no experimental procedure (e.g. randomization) was applied. The switch was independent from the decision to include the patients in this cohort, thereafter the local EC ruled out the requirement of formal ethics approval; all patients gave their informed consent uniquely for the use of clinical and laboratory data.

Once included, patients were prospectively followed accordingly to current clinical practice as indicated from Italian guidelines with visits after 2, 4 months and thereafter every 3–6 months. Each visit included a physical examination and blood analysis (including HIV-RNA) performed using standard methods.

For the only seek of this report, the primary endpoint was the virological response, defined as the proportion of patients with HIV viral load below 50 copies/ml 5 years after the switch, similarly patients with greater than > 50 copies/ml were asked to perform a new test within a months and in the case of confirmed positivity were considered virological failures.

Several other commonly collected data were used to evaluate secondary endpoints. Safety and tolerability were studied by analyzing questioning of patients at each visit and on the basis of physical examination and laboratory analysis. We evaluated immunological changes in terms of CD4+, CD8+, cell/counts and CD4/CD8 ratio variations and we also collected changes in creatinine and blood lipid content as possible markers of drug toxicity.

Data are presented as medians and interquartile range or percentages. Student’s t-test for paired samples was employed to identify significant changes in immunological, renal and metabolic functions. Kaplan-Meier curves were used to describe persistency on therapy; a Cox regression model was adopted to evaluate baseline characteristics and the risk of stopping therapy. We did all statistical analyses using SPSS version 17.

## Results

Two-hundred eighteen individuals who switched their regimen (between January 2015 and July 2016) were enrolled and prospectively followed. Table 1 summarizes baseline characteristics.

**Table 1:** Baseline characteristics of the cohort (n = 281)

Characteristics	Median (IQR) or number (%)
Age (years)	52 (47–59)
Gender Male female	211 (75.2%) 70 (24.8%)
Origin Italy Other Countries	264 (94.0%) 17 (6.0%)
Risk factor for HIV Heterosexual contacts MSM IVDU Other	129 (45.9%) 78 (27.8%) 72 (25.6%) 2 (0.7%)
Co-infection HCV Yes No	46 (16.5%) 235 (83.5%)
Time on ART (years)	10.2 (4.6–17.2)
Previous ART regimens (number)	3 (2–5)
Time with HIV-RNA < 50 copies/ml (months)	75 (33–122)
Baseline CD4 (cells/mcL)	669 (483–927)
Baseline CD8 (cells/mcL)	767 (532–1034)
CD4/CD8 ratio	0.93 (0.61–1.30)

Median age of the cohort was 52 (IQR 42–59); male individuals were 75.2% and most of individuals were of Italian origin (94%). Subjects had a long ART history (median 10 years [IQR 4.6–17.2]) and were on average on their 3rd line of therapy. Median time of viral suppression before switch was 75 months (IQR 33–122). Before switch, most of individuals (93.6%) were taking a triple-drug regimen, being the most common backbones tenofovir + emtricitabine (59.2%) or abacavir + lamivudine (27.5%). The most common anchor drugs were efavirenz (18.8%) and rilpivirine (16.5%) among NNRTIs, and either boosted darunavir (14.7%) or boosted atazanavir (14.2%) among PIs. A previous exposure to INSTI was documented in 22.5% of individuals.

The main reasons for therapeutic switch were concomitant diseases and abnormality of laboratory tests followed by drug-related adverse events or possible adverse events or a potential drug-drug interaction. Several individuals had a mix of reasons. Each patient presented a median of 2 chronic non AIDS-related co-morbidities. The most frequent co-morbidities were: bone diseases (34.4%), hypertension (30.2%), liver diseases, including end-stage liver disease (24.3%), metabolic abnormalities (21.1%) and diabetes (12.4%), cardiovascular (12.3%), renal (11.0%) and neoplastic diseases (6.4%).

Because of these pathologies, patients took a median of 2 (IQR 1–3) and up to 11 different drugs including, but not limited to, diuretics, beta-blockers, calcium-antagonists, aspirin, statins, benzodiazepines, vitamins, proton pump inhibitors, insulin, metformin.

**Fig. 1:**
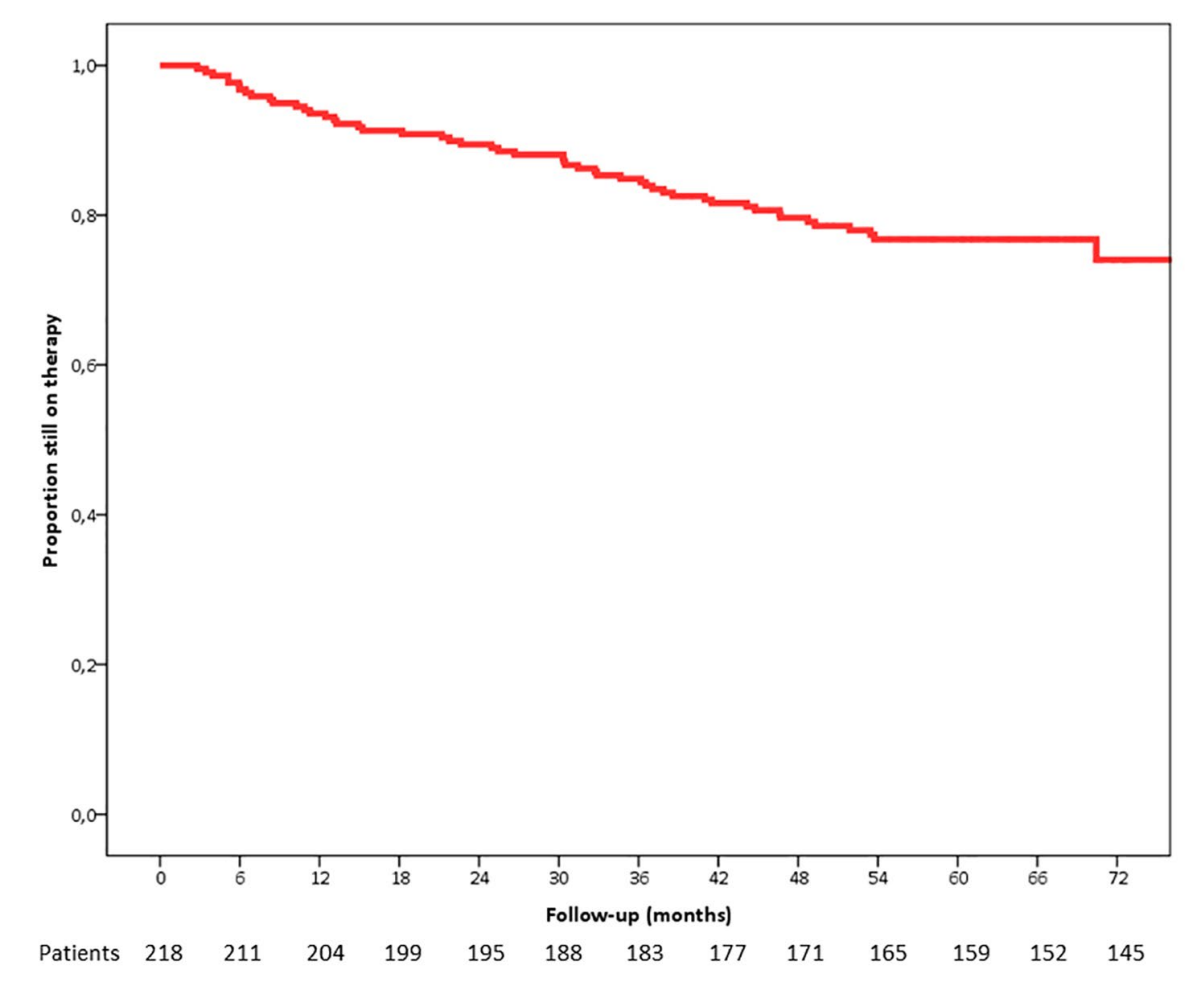
Kaplan-Meier curve representing the proportion of subjects still on the DTG-3TC combination

The mean estimated follow-up was of 64.3 months (95% CI 61.3–67.3) for approximately 1000 patient/years. After 5 years of follow-up, 77.1% were still on the DTG/3TC combination (Fig. 1). No virologic failure was detected throughout the whole study period, and only 15 subjects presented single isolated viral blips above 50 copies/ml (not confirmed). Reasons for drug discontinuations are reported in Table 2.

**Table 2:** Reasons leading to DTG/3TC discontinuation

Reason	Number
Death Neoplastic disease End-stage liver disease Myocardial infarction Sepsis Unknown case	11 6 2 1 1 1
Adverse events Weight gain Insomnia/neurological symptoms Mialgia Other	13 3 5 3 2
Drug unrelated causes Lost to follow-up Patient’s decision Moved to other center Anti-neoplastic therapy	20 10 6 3 1
Back to triple drug regimen Poor adherence Not reported	4 2 2

Most patients stopped therapy for reasons unrelated to study drugs (lost to follow-up; patients’ decision; moved to other Centers) but due to the unselected nature of the casuistry; 11 subjects died in the 5 years of follow-up mostly because of pre-existing co-morbidities (6 had neoplastic diseases and 2 had end-stage liver disease). No patient who dropped from the study for any reason had a viral load > 50 copies at the moment of withdrawal.

At the enrollment, the median CD4 count was 669 cells/mcL (IQR 483–927); after 5 years it raised to 899 cells/mcL (IQR 646–1160) (P < 0.001), without a significant change of CD8 counts that lowered from 767 cells/mcL (IQR 532–1034) to 683 cells/mcL (IQR 538–988). Consequently, the CD4/CD8 ratio varied from 0.93 (IQR 0.60–1.30) to 1.15 (IQR 0.77–1.45) (P < 0.0001). A non-significant (P = 0.320) increment of mean creatinine, 0.06 mg/dl in magnitude, was observed over the whole follow-up and was more marked in the first two months raising the baseline value 0.94 mg/dl (IQR 0.78–1.11) to 0.97 mg/dl (IQR 0.84–1.13), but thereafter leveled on these values being the median after 5 years 1.01 mg/dl (IQR 0.84–1.20). The lipid profile was not influenced by switching to the dual regimen. Median total cholesterol variation was − 10 mg/dl (from 188 mg/dl [IQR 158–217] to 178 mg/dl [IQR 162–178]); median HDL-cholesterol change was − 1 mg/dl (from 47 mg/dl [IQR 38–56] to 46 mg/dl [IQR 39–60]) and triglycerides variation was − 4 mg/dl (from 109 mg/dl, [IQR 91–138] to 105 mg/dl [IQR 85–162]).

## Discussion

With highly-effective antiretroviral drugs, the need of simpler, more manageable and less impactful regimens is becoming one of the priority in more and more patients in our clinics. From a physician’s perspective, the main consideration about whether to switch from a triple combination therapy to an equally effective dual regimen or not is the supposed possibility of reducing long-term side effects, making a pro-active switch. From the patients’ point of view, the success of 2DR in real-practice is driven also by the ease and the handling of these drugs, in particular if used as single-tablet regimens, with the least impact on their daily life [15, 18].

The indication of DTG/3TC regimen in the switching strategy is the result of randomized clinical trials (RCTs) such as the recently updated TANGO study, which supports the effective virologic suppression at 144 weeks of DTG/3TC combination, non-inferior to tenofovir alafenamide (TAF)-based ART [19], in patients without prior virologic failure, NRTI resistance, or HBV co-infection. More recently, the results of the SALSA study reinforced this indication [20].

Besides RCTs, real-life studies have gathered lots of data about the use of DTG/3TC, with interesting aspects and considerations. In particular, these data come from evaluation on patient populations that, for different reasons, would have not be considered eligible for a clinical trial but that would mostly benefit from the switch in terms of less drug-drug interactions, less toxicities, ease of use, better tolerability and, as consequence, better adherence.

A meta-analysis conducted by Patel et al. confirmed the effectiveness and safety of DTG/3TC in clinical practice, combining the data reported in several real-world studies and, therefore, supporting outcome from RCTs; in particular, virologic suppression ranged from 97 to 100% and 92–100% at 48-weeks and 96-weeks of DTG/3TC in the real-life studies considered and available so far [21]. Although virologic failure was a rare event in all these cohort analysis, they do not go beyond a follow-up of 144 weeks, making potentially valid a questioning about long-term outcomes.

Our study reports data for a follow-up over 5 years and a total drug exposure of about 1000 patient/years. In our prospective cohort study, the main reasons for DTG/3TC discontinuation were related to the worsening of pre-existing comorbidities or to drug unrelated events, rather than virologic failures or DTG/3TC-related adverse events.

These results support the hypothesis that DTG in dual-formulations has a well-known high genetic barrier to resistance development together with a high adherence rate, thus limiting the risk of virologic failure and development of resistance inducing mutations. With respect to this, to the best of our knowledge, in only one case, a new NRTI mutation (M41M/L) appeared at failure of the 2DR with DTG/3TC [18], yet with an uncertain role (if any) in the occurrence of failure itself; only recently the emergence of R263K and S230N mutations in the integrase region was detected by genotypic deep sequencing in a patient failing DTG/3TC [22].

Our study has some limitations. First, in clinically unselected uncontrolled cohorts there is always the risk of bias due to unmeasured confounding, such as drug adherence levels or not reporting adverse effects. For this reason, we decided to report only adverse events leading to drug discontinuation or to clinically relevant decision (e.g. change in therapy). Another caveat was that we censored patients who were lost to follow-up. Such censoring could have led to bias in increasing rate of treatment failure. Finally, our study results could not be applied to those health care systems that do not have HIV case management programs and free ART available.

Nevertheless, these long-term results over 5 years reinforce the durable efficacy, high barrier to treatment-emergent resistance, and good tolerability of DTG/3TC. Our results continue to support the recommended switch use of this 2DR as a well-accepted treatment option for virologically suppressed ART-experienced PLWH.

## Data Availability

The datasets generated and/or analysed during the current study are not publicly available since their containing information that could compromise the privacy of patients but are available from the corresponding author on reasonable request.

## Declarations

RG has served as consultant on advisory boards for Abbvie, Bristol-Myers Squibb, ViiV Healthcare and received lecture fees from Bristol-Myers Squibb, Janssen-Cilag, Roche, ViiV Healthcare, Merck Sharp and Dome.

CM has served as a consultant on advisory boards for Abbvie, Bristol-Myers Squibb, Gilead, ViiV, Janssen, she has received lecture fees from Bristol-Myers Squibb, Gilead,ViiV, Merck Sharp and Dome, and has received research and educational grants from Gilead and ViiV. Other authors: no conflict.

## Abbreviations

**ART**: Antiretroviral Therapy.
**HCV**: Hepatitis C Virus.
**HIV**: Human Immunodeficiency Virus.
**IVDU**: Intravenous Drug Users.
**MSM**: Men who have Sex with Men
